# Ventilator-associated events criteria in the assessment of Ventilator-Associated Pneumonia (IMPACTO MR-PAV): A prospective cohort

**DOI:** 10.1016/j.bjid.2025.104543

**Published:** 2025-05-22

**Authors:** Giovanna Marssola Nascimento, Daniela Laranja Gomes Rodrigues, Filipe Teixeira Piastrelli, Maysa Yukari Cheno, Katia Cristina Camondá Braz, Lucas Bassolli de Oliveira Alves, Bruno Martins Tomazini, Viviane Cordeiro Veiga, Beatriz Arns, Bruno Adler Maccagnan Pinheiro Besen, Antonio Paulo Nassar Junior, Alvaro Avezum, Renata Karoline Lima da Silva, Conceição de Maria Pedrozo e Silva de Azevedo, Maria Luiza Santana de Oliveira Silva, Graziela Regina Kist, Fernanda Borges Salgado, Maria Tereza Farias de Moura, Emerson Boschi, Pedro Martins Pereira Kurtz, Eva Regina Valadares da Silva Miraglia, Thawani Andrade de Lima, René Rodrigues Pereira, Jussara Alencar Arraes, Haliton Alves de Oliveira Junior

**Affiliations:** a Hospital Alemão Oswaldo Cruz, Responsabilidade Social, São Paulo, SP, Brazil; b Hospital do Coração, Instituto de Pesquisa, São Paulo, SP, Brazil; c BP ‒ A Beneficência Portuguesa de São Paulo, São Paulo, SP, Brazil; d Hospital Moinhos de Vento, Porto Alegre, RS, Brazil; e Hospital Sírio-Libanês, São Paulo, SP, Brazil; f Hospital Israelita Albert Einstein, São Paulo, SP, Brazil; g Hospital Alemão Oswaldo Cruz, Centro Internacional de Pesquisa, São Paulo, SP, Brazil; h Hospital Jean Bitar, Belem, PA, Brazil; i Hospital Presidente Vargas, Porto Alegre, RS, Brazil; j Hospital Estadual Geral de Goiânia Dr. Alberto Rassi, Goiânia, GO, Brazil; k Hospital Ernesto Dornelles, Porto Alegre, RS, Brazil; l Hospital Municipal de Maringá, Maringá, PR, Brazil; m Hospital do Tricentenário, Olinda, PE, Brazil; n Hospital Geral de Caxias do Sul, Caxias do Sul, RS, Brazil; o Instituto Estadual do Cérebro Paulo Niemeyer, Rio de Janeiro, RJ, Brazil; p Hospital Universitário de Brasília, Brasília, DF, Brazil; q Hospital AC Camargo, São Paulo, SP, RJ, Brazil; r Hospital OTO Aldeota, Fortaleza, CE, Brazil; s Hospital Maternidade São Vicente de Paulo, Barbalha, CE, Brazil

**Keywords:** Pneumonia, ventilator-associated, Ventilator-associated events, Surveillance, Healthcare-associated pneumonia

## Abstract

**Background:**

Ventilator-Associated Pneumonia (VAP) is a critical healthcare-associated infection, but no universal surveillance standard exists. In 2013, the CDC revised its criteria, incorporating Ventilator-Associated Events (VAEs) with VAPs as a subset. In Brazil, however, the Health Regulatory Agency (ANVISA) chose to retain the traditional VAP criteria. This study aimed to evaluate the incidence of VAP using both the traditional and revised criteria.

**Method:**

We conducted a prospective multicentric cohort of critically ill adult patients who required mechanical ventilation in 12 Brazilian Intensive Care Units (ICU) over six months. We evaluated the level of agreement between the two criteria considering frequency and kappa coefficient. This study was registered at ClinicalTrials.gov, NCT05589727.

**Results:**

The study included 987 patients and revealed that 85.7 % of VAP reported by the centers according to ANVISA criteria were not confirmed by the adjudicators. Among the adjudicators, a 16.7 % disagreement (kappa = 0.32) suggested subjectivity in applying VAP criteria. Between the two sets of criteria, an 11% disagreement (kappa = 0.12) was observed. However, manual adjudication of automatically generated VAEs showed only a 4 % disagreement, indicating greater objectivity in the VAE criteria. Despite the high agreement in VAE adjudication, this did not necessarily translate to a more reliable exclusion of non-events, which is essential for accurate surveillance.

**Conclusion:**

The findings highlight the challenges in identifying and classifying VAP, emphasizing the need for improved surveillance methods. The results could inform enhancements in VAP monitoring in Brazil and potentially impact other countries using similar criteria.

## Background

Healthcare-Associated Infections (HAIs) are considered adverse events. In addition to increasing hospitalization time, morbidity, and mortality in hospitals, HAIs also impose various economic impacts on healthcare systems.^1^ Ventilator-Associated Pneumonia (VAP) is a serious complication of mechanical ventilation, affecting 8 % to 28 % of ventilated patients globally.^2^, ^3^, ^4^, ^5^ The definition of infection diagnostic criteria for the epidemiological surveillance of HAIs in healthcare services allows for the necessary standardization to identify cases by healthcare professionals from different institutions systematically. However, concerning VAP, the absence of a gold standard in diagnosis complicates the proper evaluation of different case definitions. The sensitivity and specificity variables of available clinical criteria make diagnostic evaluation complex.^6^^,^^7^

The CDC (Centers for Disease Control and Prevention) criteria were revised in 2013, adopting concepts of Ventilator-Associated Events (VAEs): Ventilator-Associated Condition (VAC); Infection-Related Ventilator-Associated Complication (IVAC); Possible Ventilator-Associated Pneumonia (PVAP).^8^ In Brazil, as in other countries, the regulatory surveillance agency (ANVISA) opted not to follow the changes proposed by the CDC, considering the need to evaluate whether these changes would be feasible for Brazilian hospitals, maintaining the traditional criteria. However, given the high incidence and mortality rate of VAP, there is an urgent need for reliable surveillance methods to accurately identify and track cases, as well as for the global standardization of diagnostic criteria.

In this study, we evaluated the incidence of VAP using both VAE and VAP criteria, comparing the agreement between both criteria.

## Methods

We followed the STrengthening the Reporting of OBservational studies in Epidemiology (STROBE) statement for reporting this study.^9^

### Study design

A comprehensive description of the study's methods has been previously published.^10^ Briefly, this study involved a multicenter observational prospective cohort study carried out in approximately 15 hospitals enrolled in the IMPACTO MR platform,^11^ covering all geographical regions of Brazil. Representativeness of Brazilian ICU context was not our primary target, due to the great number and complexity of Brazilian ICUs, and also due to limited resources. Eligible hospitals were required to have an adult ICU, active Hospital Infections Control Committees (HICC) and to regularly report HAI to ANVISA. The study encompassed both public and private hospitals, incorporating a diverse range of ICU profiles. Hospitals participating in another study on HAI also funded by the Brazilian Ministry of Health (MoH) were not eligible to participate.^12^ This decision was made to mitigate potential biases since the other study has an intervention on HAIs.

Trial registration number NCT05589727; Clinicaltrials.gov.

### Eligibility

We included all patients aged 18 years or older who were hospitalized in public or private ICUs and required mechanical ventilation. We excluded all patients whose data quality could not be ensured, even after the implementation of specific training and guidelines. Additionally, we excluded records that were duplicates, incomplete, submitted after the deadline, or missing essential data.

### Data collection and source or information

Data collection spanned 6 months at each site. Given the study's accrual duration (> 1 year) and the coverage across different Brazilian regions with varying initiation dates, seasonal sampling was deemed unnecessary.

The research team from the coordinating center trained all professionals from the HICCs of each participating site to collect data. Training content comprises contextualization of the theme, justification of the project, objectives, methodology (eligibility criteria, design, data collector profile, data to be collected), presentation of the data collection system, step-by-step demonstration of data collection, data analysis, support and materials for participating centers, team documents. Only professionals who completed the training were authorized to collect data, guaranteeing consistency and reliability. All training sessions were recorded in minutes. The participating centers and the training log are available in the Supplementary Material. The research team from each center monitored patients throughout their mechanical ventilation period in the participating ICU. We captured demographic and clinical profile data through the Epimed Monitor System database, an administrative database utilized for quality improvement in Brazil upon the patients' admission to the study ICU.^13^

**Fig. 1:**
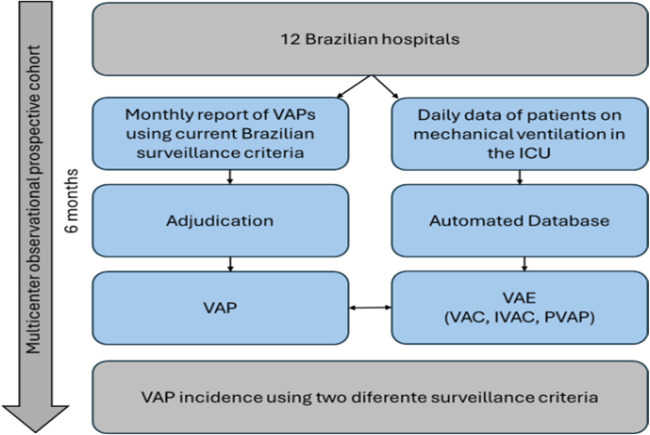
IMPACTO MR-PAV study flowchart. ICU, Intensive Care Unit; VAC, Ventilator-Associated Condition; IVAC, Infection-Related Ventilator-Associated Complication; PVAP, Possible Ventilator-Associated Pneumonia; VAE, Ventilator-Associated Events; VAP, Ventilator-Associated Pneumonia.

The trained local researchers entered mechanical ventilation data daily into the institutional database system (“VAP System”) from the initiation of mechanical ventilation until the day following its cessation. VAPs reported to ANVISA, according to its criteria, were recorded in the database system for adjudication which was carried out independently by two professionals (a physician and a nurse) with experience in HAIs surveillance. They underwent thorough training via videoconferencing and received a manual with instructions. In case of disagreement between the first and the second adjudicators, a third (physician) was consulted. We applied the CDC criteria^14^ to define VAPs, while a specific algorithm automatically provided the VAE definition in the database system. A nurse experienced in HAI surveillance manually validated the algorithm's diagnosis (Fig. 1; Supplementary Table 1).

The demographic profile of population was described, including age, sex, admission diagnosis, pre-admission origin, Simplified Acute Physiology Score-3 (SAPS3) and Charlson index.^15^^,^^16^

### Outcomes

#### Primary outcome measures

Incidence of VAP using two different surveillance criteria: The current ANVISA criteria^17^ for VAP versus the VAE criteria defined by the CDC.^14^.

#### Secondary outcome measures

1. Description of the two criteria for identifying VAP: The diagnostic of the two criteria for identifying VAP will also be compared, characterizing events associated with mechanical ventilation other than VAP, when applicable ‒ VAC, IVAC and PVAP.

2. Adjudication of VAP: To adjudicate VAP reported to ANVISA using current epidemiological diagnostic criteria

A subgroup analysis, categorized by event, was conducted to describe the following variables: days of MV, days in the ICU, days of antibiotic therapy, and deaths during MV.

The overall incidence of VAP per 1000 ventilator-days, Ventilator-Associated Conditions (VACs) per 1000 ventilator-days, Infection-related Ventilator-Associated Complications (IVACs) per 1000 ventilator-days, and Possible Ventilator-Associated Pneumonias (PVAPs) per 1000 ventilator-days was calculated. Outcome data were compared across different VAP groups. Before the start of the study, two validation tests of the VAP System were carried out. The first involved an artificial simulation of events (VACs, IVACs, and PVAPs), while the second utilized formal patient data collected at the coordinating site. These tests confirmed the correct identification of all events without ambiguous classifications. The VAP System's diagnosis underwent manual validation by a nurse experienced in HAI surveillance as a double-check.

### Statistical analysis

Initially, we planned to achieve a sample size of 226 individuals, which would allow us to detect a kappa coefficient of 0.8 (or greater) with a statistical power of 90 %. This assumption was made considering a kappa of 0.5 under the null hypothesis, a two-sided hypothesis test with a significance level of 5 %, and a range of positive evaluation proportions (positive diagnosis) spanning from 0.10 to 0.90. The kappa coefficient, along with a 95 % Confidence Interval (95 % CI), would be utilized to assess the degree of agreement between two diagnostic methods characterized by binary responses (positive or negative). Prevalence-adjusted Kappa was also applied as a sensitivity analysis once we expect low incidence of events. Agreement (kappa) for identifying VAP between the two criteria was evaluated through VAPs confirmed by adjudication (ANVISA criteria) and PVAPs validated in automated surveillance (CDC criteria). The interpretation of agreement would adhere to the Landis and Koch criteria, categorizing agreement as almost perfect for values from 0.81 to 1.00, substantial for values from 0.61 to 0.80, moderate for values between 0.41 and 0.60, fair for values between 0.21 and 0.40, slight for values from 0 to 0.20, and poor for negative values.^18^ Furthermore, if the sample size were less than 30, the CI for kappa would be estimated using the bootstrap method. However, upon reaching the estimated sample size in November 2022, only a few VAP events had been reported. Consequently, drawing reliable conclusions from such a limited event rate was deemed unfeasible. As a result, the IMPACTO MR-PAV steering committee opted to extend recruitment and recalculate the sample size based on VAP incidence. The redefined sample size was set to 1117 participants. This would lead to a sample size of 22 cases and 718 controls (Supplementary Material). As we are dealing with imperfect standard tests, we compared the two criteria using two approaches: 1) Narratively, by discussing the event rate and qualitative results with both tests; 2) By the Kappa coefficient^19^ with a 95 % Confidence Interval (95 % CI) was used to assess the degree of agreement between two diagnostic methods, defined by binary responses (positive or negative event). Initially, we have planned to use a Bayesian approach to specifically evaluate the diagnostic accuracy between the two methods. However, considering the anticipated disagreement rate for positive results between the tests (i.e., VAP agreement), the limitation in the outcome assessment (i.e. while the Anvisa criterion is binary (VAP vs. No event), the CDC criteria have more than one category), and considering that CDC criteria also assumes independence of observations, (that is, re-events should be excluded from the model) such a test is not worthwhile and is not very informative. The Kaplan-Meier method was applied to describe event incidence according to the CDC criteria. Data analysis was performed using RStudio 12.0 (RStudio Team, Boston, MA).^20^ All hypothesis tests were two-sided, and a p-value < 0.05 was considered statistically significant.

## Results

In the beginning, 14 centers achieved the proper feasibility to participate in the study and were invited to the site initiation visit. However, two of then dropped out before the study starts. The exclusion reasons for both centers were related to contractual and technical aspects (Difficulties in the agreement signing, change of PI, lack of response). Therefore, the first 12 hospitals that met the inclusion criteria and expressed interest in the study were selected. The list of participating centers and the reasons for exclusion are available in the Supplementary Material. Table 1 and Supplementary Figure 1 characterizes the sample of 987 patients (1115 ventilation episodes) included in the study. Until the end of the study, 57 ventilation episodes were excluded from the main analysis: 15 could not have data quality guaranteed by the participating site even despite various training and guidelines, and 42, were incorrect registries in the VAP-System, after the deadline, in duplicate or with essential missing data.

**Table 1:** Sample characterization.

Category	Total (n = 987)
Age, median (Q1‒Q2)	66.2 (53.5‒75.6)
Male, n (%)	578 (51.8)
Primary Admission Diagnosis, n (%)	
- Infection	307 (27.5)
- Surgical / Intervention	247 (22.2)
- Cardiologic	111 (10.0)
- Neurologic	93 (8.3)
- Others	352 (31.6)
Pre-admission Origin, n (%)	
- Home	475 (45.9)
- Hospital / Health service	355 (34.3)
- Emergency Unit	172 (16.6)
- Backup Hospital	14 (1.4)
- Nursing home	10 (1.0)
- Homecare	8 (0.8)
Charlson Index (points), median (Q1‒Q3)	1 (0‒3)
SAPS (points), median (Q1‒Q3)	62 (48‒73)

Missing data: Pre-admission Origin (n = 81); Charlson index (n = 69); SAPS (n = 56); Primary Admission Diagnosis (n = 5).

The VAP System identified 66 VAEs according to CDC criteria. The incidence density was of 6.7 VAEs per 1000 ventilator days. The identified events are as follows 41 VACs (4.1 VACs per 1000 ventilator days); 19 IVACs (1.9 IVACs per 1000 ventilator days); 6 PVAPs (0.6 PVAPs per 1000 ventilators day). The sites reported 84 VAPs according to ANVISA criteria. The incidence density was of 8.5 VAPs per 1000 ventilator days.

The agreement between adjudicators and VAPs reported by the participating sites according to ANVISA criteria was evaluated. Therefore, 85.7 % of VAPs reported by the centers according to ANVISA criteria were not confirmed by the adjudicators (Table 2) (Supplementary Fig. 2). Furthermore, the reasons for non-confirmation by the adjudicators were: criteria outside the infection window period (5 reported VAPs); absence of mechanical ventilation criteria for definition (9 reported VAPs); absence of radiological worsening (23 reported VAPs); and absence of clinical/laboratory criteria (14 reported VAPs) (Supplementary Fig. 2). A kappa of 0.32 (95 % CI 0.0; 0.6) was found among the adjudicators (Table 2).

**Table 2:** Agreement between methods.

Agreement Between Methods	n	Kappa (95% CI)	Prevalence-adjusted Kappa (95% CI)	Disagreement Rate
Agreement between adjudicators (VAP ANVISA)	84	0.32 (0.0-0.6)	0.66 (0.47-0.81)	14 (16.7%)
Adjudicator-criterion ANVISA agreement	84	⁎	⁎	72 (85.7%)
System-Nurse (CDC) agreement	1115	0.69 (0.6-0.8)	0.91 (0.89-0.94)	45 (4.0%)
ANVISA-CDC agreement	1115	0.12 (0.0-0.2)	0.78 (0.74-0.81)	123 (11.0%)

⁎ In this case only the Anvisa criteria was evaluated, therefore kappa was not calculated.

Finally, an analysis of agreement between ANVISA (VAP) and CDC (VAE) criteria was performed. There was disagreement in 123 cases. For the remaining 992 cases, the criteria agreed that there was no event, resulting in a discordance rate of 11 %. Manual adjudication (by a nurse with expertise in infection control) was also performed for the VAEs automatically generated by the VAP System. From these data, it was possible to calculate the agreement between manual and automated reporting of VAEs (CDC criteria). A kappa of 0.69 (95 % CI 0.6; 0.8) was found. Prevalence-adjusted Kappa showed higher values for agreement than the not adjusted Kappa, independently of the comparison (Table 2).

Outcomes of mechanically ventilated patients according to the occurrence of events, including the duration of ventilation, ICU stay, duration of antibiotic use, and mortality, are detailed in Table 3. Of the 1115 records analyzed, the overall mean ventilation duration was 8.9 ± 9.9 days, mean ICU stay was 17.3 ± 20.3 days, and mean antibiotic use was 7.0 ± 6.7 days. The death rate during intubation was 37.0 %. For patients with no events recorded according to the CDC criteria, the mean ventilation duration was 8.3 ± 9.7 days, ICU stay was 17.1 ± 20.5 days, and antibiotic use was 6.5 ± 6.3 days, with a death rate of 35.4 %. Similar trends were observed for patients with no events recorded according to ANVISA criteria.

**Table 3:** Outcomes according to the occurrence of events.

Total	n	Ventilation days	ICU Days	Antibiotic Days	Death during mechanical ventilation (%)
Total	1115	8.9 ± 9.9	17.3 ± 20.3	7.0 ± 6.7	413 (37.0 %)
No event records (CDC)	1049	8.3 ± 9.7	17.1 ± 20.5	6.5 ± 6.3	371 (35.4 %)
No event records (ANVISA)	1032	8.2 ± 9.4	16.4 ± 19.5	6.5 ± 6.4	378 (36.6 %)
Event records (criteria intersection	13	23.2 ± ± 11.0	28.0 ± 14.7	20.4 ± 10	6 (46.2 %)
Event records (ANVISA)	83	17.7 ± 12.1	29.5 ± 25.4	13.2 ± 7.9	35 (42.2 %)
Event records (CDC)	66	17.5 ± 10.2	22.0 ± 16.4	14.9 ± 8.9	42 (63.6 %)
VAC (CDC)	41	16.1 ± 9.7	20.5 ± 17.0	13.7 ± 7.7	28 (68.3 %)
IVAC (CDC)	19	19.2 ± 12.1	22.4 ± 14.4	16.5 ± 11.9	12 (63.1 %)
PVAP (CDC)	6	21.0 ± 4.0	32.0 ± 18.3	17.3 ± 4.1	2 (33.3 %)

Values are presented as mean ± SD. (%) or absolute numbers and percentages.

ICU, Intensive Care Units; VAE, Ventilator-Associated Events; VAC, Ventilator-Associated Condition; IVAC, Infectious Ventilator-Associated Condition; PVAP, Possible Ventilator-Associated Pneumonia; CDC, Centers for Disease Control; ANVISA, Agência Nacional de Vigilância Sanitária.

Patients with events recorded based on the intersection of both criteria had significantly higher mean ventilation duration (23.2 ± 11.0 days), ICU stay (28.0 ± 14.7 days), and antibiotic use (20.4 ± 10 days), with a death rate of 46.2 %. This indicates that patients with overlapping criteria for events experienced more severe outcomes. Among the ANVISA event records, patients had a mean ventilation duration of 17.7 ± 12.1 days, ICU stay of 29.5 ± 25.4 days, and antibiotic use of 13.2 ± 7.9 days, with a death rate of 42.2 %. Similarly, CDC event records showed a mean ventilation duration of 17.5 ± 10.2 days, ICU stay of 22.0 ± 16.4 days, and antibiotic use of 14.9 ± 8.9 days, with a higher death rate of 63.6 %.

Specific events categorized under CDC criteria, such as VAC, IVAC, and PVAP, also showed worse outcomes. Patients with VAC had a mean ventilation duration of 16.1 ± 9.7 days, ICU stay of 20.5 ± 17.0 days, and antibiotic use of 13.7 ± 7.7 days, with a death rate of 68.3 %. IVAC patients had higher ventilation duration (19.2 ± 12.1 days), ICU stay (22.4 ± 14.4 days), and antibiotic use (16.5 ± 11.9 days), with a death rate of 63.1 %. PVAP patients experienced the highest mean ventilation duration (21.0 ± 4.0 days), ICU stay (32.0 ± 18.3 days), and antibiotic use (17.3 ± 4.1 days), but a lower death rate of 33.3 % (Table 3).

It was observed that the incidence of VAP increased from 8 % (95 % CI: 6 %‒11 %) at 10 days to 17 % (95 % CI: 13 %‒23 %) at 30 days and 20 % (95 % CI: 14 %‒28 %) at 60 days (Supplementary Fig. 3). The main clinical outcomes and their frequency are reported on Table 3.

**Fig. 2:**
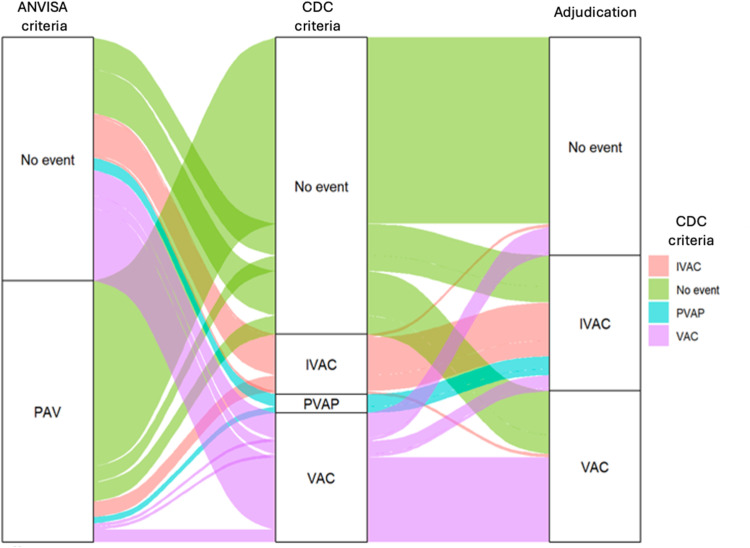
Sankey diagram comparing ANVISA criteria, automated CDC criteria and adjudication.

The Sankey diagram (Fig. 2) visually represents the flow of mechanically ventilated patients through different stages of Ventilator-Associated Events (VAEs) and Ventilator-Associated Pneumonia (VAP), as assessed by both the ANVISA and CDC criteria. This diagram highlights the progression and overlap between various event categories such as VAC, IVAC and PVAP. The varying thickness of the lines reflects the proportion of patients moving from one event status to another. Notably, the diagram reveals significant transitions from “No Event” to other VAE categories.

## Discussion

The findings of this study reveal significant challenges in identifying and classifying VAPs. The discrepancy between the ANVISA criteria and the CDC's VAEs criteria, as well as between the adjudicators themselves, highlights the subjectivity and complexity of the current criteria. The analysis demonstrated that the agreement rate was substantially higher for automatically generated VAEs, suggesting greater objectivity in the CDC criteria.

The incidence density was 6.7 VAEs per 1000 ventilator days in this study. Regarding VAP according to ANVISA criteria, the incidence density was 8.5 VAPs per 1000 ventilator days. Several previous studies have reported the incidence of VAEs. However, the data are very heterogeneous.^21^, ^22^, ^23^, ^24^, ^25^ The incidence rates reported to the CDC for the first full year of VAE surveillance (2014) ranged from 2.59 to 11.79 per 1000 ventilator days, with higher rates found in university hospitals.^26^ Lower rates were reported in a study of 7 urban hospitals in Japan (6.4 VAEs per 1000 ventilator days),^27^ and higher rates were observed in a multinational cohort in Europe (40.8 VAEs per 1000 ventilator days).^28^ A study with more than 6000 ventilated patients in five medical and surgical specialty ICUs at an academic medical center in China reported VAC, IVAC, and PVAP rates of 13.7, 6.3, and 2.2 per 1000 ventilator days, respectively.^29^

Regarding traditional VAP criteria (ANVISA), the incidence density was 8.5 VAPs per 1000 ventilator days. Data published by the Epidemiological Surveillance Center of the State of São Paulo indicated 7.08 VAPs per 1000 ventilator days (p50) in adult ICUs of Public Hospitals in 2022.^30^ The 2022 national report from ANVISA indicates 9 VAPs per 1000 ventilator days (p50) in adult ICUs in Brazil.^31^. Thus, the data from the study in question is quite consistent with recent reports.

When we analyze the agreement between the two criteria, the result of 11 % discordance is justified because they are fundamentally different criteria – although both are used for VAP. VAE was intentionally designed to be different from the traditional VAP criterion. The VAE requirement for a sustained increase in ventilator settings creates a threshold effect that selects patients with severe illness.^5^

The new VAE surveillance detected only one-third of conventional VAP cases (among the 165 reported) in a previous study.^32^ A meta-analysis and systematic review evaluated 18 studies with 61,489 patients receiving mechanical ventilation in ICUs across eight countries and identified that the combined sensitivity and positive predictive value of each type of VAE for detecting VAP did not exceed 50 %, while the combined specificity and negative predictive value exceeded 80 %. It was concluded that VAE surveillance performs well in the absence of a case but does not accurately detect traditional VAP cases in ICUs.^33^

The high rates of disagreement found in the ANVISA criteria are supported by studies indicating significant interobserver variability in VAP surveillance, reflecting the challenges in consistently applying subjective criteria.^7^^,^^30^ One study sought to evaluate interobserver variability in VAP surveillance. Three infection control professionals and one physician independently evaluated 50 patients with respiratory deterioration through retrospective chart reviews. The three reviewers agreed on only 7 VAPs (kappa = 0.40). It was concluded that interobserver variability in assessing ventilator-associated pneumonia is high.^34^ In another study, investigators distributed 6 case vignettes to 43 infection control specialists in the United States. An almost equal number of respondents considered that 1, 2, 3, 4, or 5 of the 6 patients met the VAP criteria.^35^.

Other reasons can also explain this variation. Firstly, we must consider the limitation of the method used for adjudication. The data were entered into the VAP System by the notifier. The adjudicator did not have access to the full patient's medical records, unlike those who reported the events. And, even for reported events, the adjudicators only had access to patient’s medical records the site selected to attach to the VAP System. Another challenge was about sites where the medical records were physical. This made it difficult to access some information on time. This could be considered a potential information bias and a restriction in the comprehensive evaluation of each case. Third, another possible reason for the discrepancies is the heterogeneity of Hospital Infection Control Committees (HICCs) in Brazil evidenced through a National Assessment of Health Service Infection Prevention and Control Programs conducted by ANVISA in 2023. In the evaluation of 1977 services, although 91.40 % reported having doctors and nurses on the HICC team, it was also reported that 23.58 % of these professionals did not have specific training in infection prevention and control.^36^ During the conduction of our study, it was possible to observe the discrepancy between different public and private participating sites regarding location, team size, professional expertise, accessibility and quality of medical record keeping (physical or electronic), and scientific maturity. This disparity may have directly interfered with data collection, making it difficult at times. Additionally, despite the sample size adjustment, due to the low event rate, the subjectivity observed in interrater agreement, and the presence of imperfect standards, we decided not to conduct a formal diagnostic accuracy evaluation. Therefore, we aborted the Bayesian analysis and focused in a more descriptive analysis.

Lastly, the agreement between the system and nurse (CDC) was substantial, with a Kappa value of 0.69 (95 % CI: 0.6‒0.8) and a low discordance rate of 4.0 %. This indicates a high level of consistency between the system-generated assessments and those conducted by nurse, suggesting reliability in the CDC method. This reflects the objectivity of the criteria, allowing for standardization of surveillance, which is not dependent on the examiner. We might observe an increased Kappa Index after prevalence adjustment. Nevertheless, prevalence-adjusted Kappa is not free of bias. Simulation studies indicates that it would result in substantial overestimation of reliability.^37^

The VAE algorithm's diagnostic process is automated, and manual adjudication was performed by a single nurse as a secondary verification step. Since the ANVISA criteria are examiner-dependent and require manual evaluation, the adjudication process for this method was more robust. In contrast, the VAE criteria are originally designed for automation, which is why we developed a platform to standardize and automate the process. The manual review by the nurse served only as a double-check to ensure data accuracy. Furthermore, two validation steps were conducted with VAE algorithm before its implementation: The first involved an artificial simulation of events (VACs, IVACs, and PVAPs), while the second utilized formal patient data collected at the coordinating site. These tests confirmed the correct identification of all events without ambiguous classifications. Given these differences, we acknowledge that variations in the adjudication process may not impact the comparison of agreement between the two methodologies. We believe that applying a manual adjudication process similar to ANVISA for the VAE algorithm would not significantly alter the results, as the core of the VAE methodology relies on automated detection rather than subjective interpretation.

Finally, when we evaluate the outcomes of the events, for records with no event identified by CDC or ANVISA criteria, the mean duration of ventilation, ICU stay, and duration of antibiotic use were similar. However, for records identified as events by ANVISA, CDC, or by the intersection of the criteria, a higher mean duration of ventilation, ICU stay, and duration of antibiotic use were observed, along with a significantly higher mortality rate. These results highlight the association between the occurrence of adverse events and unfavorable outcomes in mechanically ventilated patients and are supported by previous studies in the literature.^25^^,^^29^^,^^32^^,^^38^

In conclusion, the results highlight the challenges in identifying and classifying VAPs and VAEs, as well as their association with potentially unfavorable outcomes in mechanically ventilated patients. Regardless of the criteria adopted for identifying these events and despite their discrepancies, these are potentially preventable scenarios that worsen patient outcomes. Therefore, the necessity of preventive measures and effective management protocols is emphasized to reduce their incidence and consequently improve clinical outcomes. Despite this research tried to avoid lack of representativeness by addressing ICUs from all the five macroregions in Brazil, some clinical and administrative factors could have impaired its full generalization. Limitations regarding hospital sampling could comprise: lack of Hospital Infection Control Committee specialized personnel, variability in healthcare quality standards, variability in ICU diaries completeness, heterogeneity in clinical research knowledge, Anvisa VAP reporting capacity, different nosocomial profile, ICU size and complexity. Our study provides valuable insights into the challenges and potential solutions for VAP surveillance in Brazil. Based on the above data, we can infer the need to revisit the identification and impact of VAPs and VAEs in patients admitted to public and private hospitals in Brazil.

## Ethics

The local Institutional Review Board (IRB) approved this study (CAAE: 52354721.0.1001.0070). All national ethical requirements were met to support this research. The IRB waived the need for informed consent for retrospective data collection (ICU medical records). This study was registered in clinicaltrials.gov (NCT05589727). The study was first registered on 21 October 2022.

## Data availability statement

Anonymized raw data can be provided after reasonable request to the authors**.**

## Authors’ contributions

HAOJ, AA and GMN conceived and designed the study, GMN, DLGR, MYC and KCCB drafted the manuscript, which was edited and revised by all authors. GMN wrote the first draft of the report with input from DLGR and HAOJ. MYC, and KCCB made a substantial contribution to the conception and the design of the manuscript. LBOA was responsible for the statistical analysis. DLGR, GMN, HAOJ, have directly accessed and verified the underlying data reported in the manuscript. All authors had full access to all the data in the study, read and approved the final version of the manuscript to submit for publication. HAOJ was responsible for the decision in submitting this manuscript.

## Funding

The study received funding from the Unified Health System ‒ Institutional Development Support Program (PROADI-SUS) of the Ministry of Health of Brazil. The sponsor had no role in study design, data collection, data analysis, data interpretation, or writing of the report.

## Conflicts of interest

The authors declare no have conflicts of interest.

## Appendix Supplementary materials

Image, application 1
