# Supplementary material for: Ventilator-associated events criteria in the assessment of Ventilator-Associated Pneumonia (IMPACTO MR-PAV): A prospective cohort
Source: Braz J Infect Dis. 2025 May 22;29(4):104543. doi: 10.1016/j.bjid.2025.104543 (PMC12150031; doi:10.1016/j.bjid.2025.104543)
Supplement: Supplementary file 1 [file mmc1.docx]

BJID-D-25-00004

**Supplementary material**

**Ventilator-associated events criteria in the assessment of Ventilator-Associated Pneumonia (IMPACTO MR-PAV): a prospective cohort**

**SUMMARY**

| IMPACTO-MR | 2 |
| --- | --- |
| IMPACTO-MR-PAV | 3 |
| LIST OF STUDY SITES | 4 |
| OUTCOME DEFINITION | 6 |
| SAMPLE SIZE REDEFINITION | 10 |
| PARTICIPATING CENTERS – EXCLUSION REASONS AND TRAINING LOG. | 11 |
| SUPPLEMENTARY TABLES | 12 |
| SUPPLEMENTARY FIGURES | 13 |
| REFERENCES | 14 |

**1 - IMPACTO-MR**

The IMPACTO-MR program is developed and coordinated in a partnership between the hospitals members of the Program to Support Institutional Development of Universal Health System (Programa de Apoio ao Desenvolvimento Institucional do Sistema Único de Saúde - PROADI-SUS): Hospital Alemão Oswaldo Cruz (HAOC), Hospital Israelita Albert Einstein (HIAE), Hospital Moinhos de Vento (HMV), Hospital Sírio-Libanês (HSL), and HCor-Hospital do Coração (IP-HCor) in a collaboration with the Brazilian Research in Intensive Care Network (BRICNet) and is supported and overseen by the Department of Science and Technology from the Brazilian Ministry of Health (DECIT/ SCTIE/MS) and by the General Management of Health Technologies of the Brazilian Health Regulatory Agency (Gerência Geral de Tecnologias em Saúde da Agência Nacional de Vigilância Sanitária - GGTES/ANVISA). In 2022, BP - A Beneficência Portuguesa de São Paulo joined the other hospitals in coordination with the project. The project is funded by the PROADI-SUS, a nationwide program aimed at strengthening and qualifying the Brazilian Universal Health System (SUS) throughout the country. The program is developed as a prospective, multicentric platform study where participating ICUs would collect data on all admitted adult patients (≥ 18 years old) on a specific data capture system that constitutes the study’s core database. This core database provides data to prospective observational studies within the platform, and each database might have specifically designed additional databases as needed.[[1]](https://paperpile.com/c/wOQJDV/zgKI)

| **Principal Investigator** | **Hospital** |
| --- | --- |
| Giovanna Marssola Nascimento | Hospital Alemão Oswaldo Cruz |
| Bruno Tomazini | Hospital Sírio Libanês |
| Adriano José Pereira | Hospital Albert Einstein |
| Alexandre Biasi Cavalcanti | Hospital do Coração |
| Alexandre Prehn Zavascki | Hospital Moinhos de vento |
| Viviane C Veiga | Hospital Beneficiência Portuguesa |

**2 - IMPACTO-MR-PAV**

IMPACTO MR-PAV is a multicentre observational study within the platform IMPACTO-MR which evaluated the incidence of VAP using the current ANVISA (Brazilian Health Regulatory Agency) criteria versus VAE criteria defined by the US National Healthcare Safety Network (NHSN)-CDC criteria. Hospital Alemão Oswaldo Cruz (HAOC) was responsible for coordinating the study.[[2]](https://paperpile.com/c/wOQJDV/j0vN)

Research team:

- Giovanna Marssola Nascimento
- Daniela Laranja Gomes Rodrigues
- Filipe Teixeira Piastrelli
- Maysa Yukari Cheno
- Katia Cristina Camondá Braz
- Lucas Bassolli de Oliveira Alves
- Lucas Salomão
- Cícera Gabriela da Silva
- Gabriel Castro
- Rosa Camila Luccheta
- Álvaro Avezum Júnior
- Haliton Alves De Oliveira Junior

**3 - LIST OF STUDY SITES**


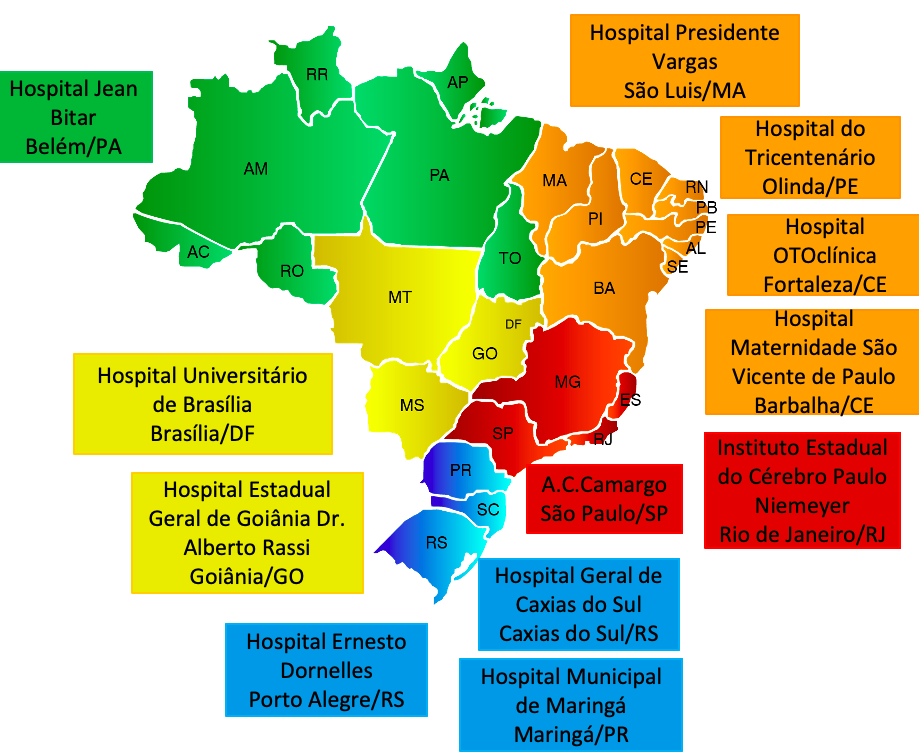


| **Principal Investigator** | **Hospital** |
| --- | --- |
| Alexandra dos Santos do Carmo | Hospital Jean Bitar |
| Elciney Cardoso Coelho | Hospital Presidente Vargas |
| Weidson Francisco Gonçalves Dantas | Hospital do Tricentenário |
| Danilo Amancio Campos | Hospital Otoclínica |
| Meton Soares de Alencar Filho | Hospital e Maternidade São Vicente de Paulo |
| Valéria Lima Paes | Hospital Universitário de Brasília |
| Amanda Carrijo Santos | Hospital Estadual Geral de Goiânia Dr. Alberto Rassi |
| Ivan Leonardo Avelino França e Silva | A.C.Camargo |
| Pedro Martins Pereira Kurtz | Instituto Estadual do Cérebro Paulo Niemeyer |
| Emerson Boschi da Silva | Hospital Geral de Caxias do Sul |
| Graziela Regina Kist | Hospital Ernesto Dornelles |
| Francielle Constantino Pereira | Hospital Municipal de Maringá |

**4 - OUTCOME DEFINITION**

1. **​​VAP surveillance in Brazil - ANVISA criteria** [[3]](https://paperpile.com/c/wOQJDV/2R3Z)

1. Patient on mechanical ventilator (MV) for a period longer than two consecutive days (i.e., starting from day 3, where day 1 is the day of MV installation) and who, on the date of infection, was either using MV or had it removed the day before.

AND

2. Without underlying heart or lung disease, with ONE or more serial imaging exams showing any of the following findings, whether new, persistent, or progressive:

· Infiltration

· Opacification/consolidation

· Cavitation

· Pneumatocele

AND

3. At least ONE of the following signs or symptoms:

· Fever (temperature: > 38ºC) without any other associated cause.

· Leukopenia (< 4000 cells/mm3) or leukocytosis (>12000 cells/mm3 or ≥ 15000 cells/mm³ with left shift and more than 10% of immature neutrophils in children ≤ 14 years old).

· Change in level of consciousness without any other apparent cause in patients ≥ 70 years old.

AND

4. At least TWO of the following signs or symptoms:

· Onset of purulent secretion or change in characteristics of the secretion or increase in respiratory secretion or increase in the need for suctioning.

· Apnea, tachypnea, dyspnea, or cough (new episode or worsening).

· Auscultation with wheezing, snoring, or crackles (new episode or worsening).

· Worsening of gas exchange, desaturation, increased oxygen demand or increased respiratory rate or changes in ventilatory parameters for at least 2 days.

AND

· The signs/symptoms and imaging findings occurred during the window period of infection.

OR

5. ONE of the criteria above (Item 4) AND at least ONE of the following results:

· Positive blood culture4 without another focus of infection.

· Positive culture of pleural fluid.

· Quantitative culture positive for pulmonary secretion obtained by a procedure with minimal potential for contamination (bronchoalveolar lavage, protected brush and endotracheal aspirate5).

· In bacterioscopy of bronchoalveolar lavage, finding of ≥ 5% of leukocytes and macrophages containing microorganisms (presence of intracellular bacteria).

· Positive culture of lung tissue.

· Histopathological examination showing at least one of the following evidences of pneumonia:

· Formation of abscess or consolidation focus with polymorphonuclear infiltration in bronchioles and alveoli;

· Evidence of invasion of lung parenchyma by hyphae or pseudo-hyphae.

· Virus, Bordetella, Legionella, Chlamydophila, or Mycoplasma identified from secretion or lung tissue culture, or identified by microbiological testing performed for clinical diagnosis or treatment purposes.

· 4-fold increase in IgG values in serology for pathogen (example: Chlamydophila).

· 4-fold increase in IgG values in serology for Legionella pneumophila serogroup I titrated ≥ 1:128 in acute and convalescent phases by indirect immunofluorescence.

· Detection of antigen of Legionella pneumophila serogroup I in urine.

AND

6. The signs/symptoms and imaging/laboratory tests occurred during the window period of infection.

1. **VAE surveillance in USA – NHSN criteria**[[4]](https://paperpile.com/c/wOQJDV/8IPu)

VAEs are identified by using a combination of objective criteria: deterioration in respiratory status after a period of stability or improvement on the ventilator, evidence of infection or inflammation, and laboratory evidence of respiratory infection. There are three definition tiers within the VAE algorithm: 1) Ventilator-Associated Condition (VAC); 2) Infectionrelated Ventilator-Associated Complication (IVAC); and 3) Possible VAP (PVAP).

1. Ventilator-Associated Condition (VAC):

Patient has a baseline period of stability or improvement on the ventilator, defined by ≥ 2 calendar days of stable or decreasing daily minimum* FiO2 or PEEP values. The baseline period is defined as the 2 calendar days immediately preceding the first day of increased daily minimum PEEP or FiO2.

* Daily minimum defined by lowest value of FiO2 or PEEP during a calendar day that is maintained for > 1 hour

AND

After a period of stability or improvement on the ventilator, the patient has at least one of the following indicators of worsening oxygenation:

· Increase in daily minimum FiO2 of ≥ 0.20 (20 points) over the daily minimum FiO2 of the first day in the baseline period, sustained for ≥ 2 calendar days.

· Increase in daily minimum PEEP values of ≥ 3 cmH2O over the daily minimum PEEP of the first day in the baseline period†, sustained for ≥ 2 calendar days.

2. Infection-related Ventilator-Associated Complication (IVAC):

Patient meets criteria for VAC AND on or after calendar day 3 of mechanical ventilation and within 2 calendar days before or after the onset of worsening oxygenation, the patient meets both of the following criteria:

· Temperature > 38 °C or < 36°C, OR white blood cell count ≥ 12,000 cells/mm3 or ≤ 4,000 cells/mm3.

AND

· A new antimicrobial agent is started and is continued for ≥ 4 qualifying antimicrobial days (QAD).

Possible Ventilator-Associated Pneumonia (PVAP)

Patients meet criteria for IVAC AND On or after calendar day 3 of mechanical ventilation and within 2 calendar days before or after the onset of worsening oxygenation, ONE of the following criteria is met:

Criterion 1: Positive culture of one of the following specimens, meeting quantitative or semi-quantitative thresholds without requirement for purulent respiratory secretions:

• Endotracheal aspirate, ≥ 105 CFU/ml or corresponding semi-quantitative result

• Bronchoalveolar lavage, ≥ 104 CFU/ml or corresponding semi-quantitative result

• Lung tissue, ≥ 104 CFU/g or corresponding semi-quantitative result

• Protected specimen brush, ≥ 103 CFU/ml or corresponding semi-quantitative result

Criterion 2: Purulent respiratory secretions (defined as secretions from the lungs, bronchi, or trachea that contain ≥ 25 neutrophils and ≤ 10 squamous epithelial cells per low power field [lpf, x100]) PLUS organism identified from one of the following specimens (to include qualitative culture, or quantitative/semi-quantitative culture without sufficient growth to meet Criterion #1):

• Sputum

• Endotracheal aspirate

• Bronchoalveolar lavage

• Lung tissue

• Protected specimen brush

Criterion 3: One of the following positive tests:

• Organism identified from pleural fluid (where specimen was obtained during thoracentesis or within 24 hours of chest tube placement; pleural fluid specimens collected after a chest tube is repositioned or from a chest tube in place > 24 hours are not eligible for PVAP)

• Lung histopathology, defined as: 1) abscess formation or foci of consolidation with intense neutrophil accumulation in bronchioles and alveoli; 2) evidence of lung parenchyma invasion by fungi (hyphae, pseudohyphae, or yeast forms); 3) evidence of infection with the viral pathogens listed below based on results of immunohistochemical assays, cytology, or microscopy performed on lung tissue

• Diagnostic test for Legionella species

• Diagnostic test on respiratory secretions for influenza virus, respiratory syncytial virus, adenovirus, parainfluenza virus, rhinovirus, human metapneumovirus, coronavirus.

**5 – SAMPLE SIZE REDEFINITION**

Upon reaching the estimated sample size in November 2022, only a few VAP events had been reported. Consequently, drawing reliable conclusions from such a limited event rate was deemed unfeasible. As a result, the IMPACTO MR-PAV steering committee opted to extend recruitment and recalculate the sample size based on VAP incidence. Typically, the literature reports VAP incidence rates ranging from 5% to 40%. Anticipating a VAP infection frequency of 3%, with a margin of error of 1% and a 95% CI, we recalculated the sample size using the equation:

n = [EDFF ∗ Np(1 − p)]/[(d2/z21 −α/2 ∗ (N − 1) + p ∗ (1 − p))]

were

EDFF = design effect (value equal to 1 was considered)

d = confidence limits

p = hypothesized% frequency of outcome factor in the population

N = population size (value equal to 1000000 was adopted).

The required sample size was determined to be 1117 participants, which would be adequate even for assessing diagnostic accuracy. For instance, assuming an area under the curve of 0.7 between tests, with an alpha of 5%, a statistical power of 90%, and the same VAP rate of 3%, this would lead to a sample size of 22 cases and 718 controls.

**6 – PARTICIPATING CENTERS – EXCLUSION REASONS AND TRAINING LOG.**

| **Training date** | **Participating center** | **Type** |
| --- | --- | --- |
| 04/10/2022 | Instituto Estadual do Cérebro Paulo Niemeyer Hospital Municipal de Maringá Hospital do Tricentenário Hospital Estadual Geral de Goiânia Dr. Alberto Rassi Hospital Regional Público do Leste de Paragominas* | Colective |
| 05/10/2022 | Hospital Geral de Caxias do Sul Hospital Presidente Vargas Hospital Estadual Geral de Goiânia Dr. Alberto Rassi | Colective |
| 06/10/2022 | Hospital Aviccena* Hospital Ernesto Dornelles Hospital Geral de Caxias do Sul Hospital Presidente Vargas Hospital Estadual Geral de Goiânia Dr. Alberto Rassi | Colective |
| 26/01/2023 | Hospital Jean Bitar | Individual |
| 02/02/2023 | Hospital Universitário de Brasília | Individual |
| 03/02/2023 | Hospital OTO clínica (OTO Aldeota) | Individual |
| 24/03/2023 | AC Camargo | Individual |
| 16/08/2023 | Hospital Maternidade São Vicente de Paulo | Individual |

*Hospital Regional Público do Leste de Paragominas was excluded due to slowness in the contract process and PI changing. After some continuos contact from the coordinating center we have not heard from them. Hospital Aviccena was excluded due to lack of contract signing, even after several attempts to contact them.

**7 – SUPPLEMENTARY TABLES**

Supplementary Table 1 – VAP and VAE criteria

| **VAP-Brazilian surveillance criteria (ANVISA)** | **VAE-USA surveillance criteria (CDC)** |
| --- | --- |
| **Subjective analysis (e.g. change in character of sputum)** | Objective analysis (e.g. PEEP, FiO2) |
| **Manual** | Potentially automatable |
| **Identifies only VAP** | Identifies other complications related to mechanical ventilation (VAC, IVAC, PVAP) |
| **Require radiographical findings of pneumonia (radiology interpretation)** | Not require radiographical findings of pneumonia |
| **Microbiological criterion is not essential to definition** | Mandatory microbiological criterion to define PVAP |
| **Antimicrobial use is not present in the criteria** | Antimicrobial use is present in the criteria |

PEEP: Positive end-expiratory pressure; FiO2: fraction of inspired oxygen; VAP: ventilator associated pneumonia; VAE: ventilator-associated events; VAC: ventilator-associated condition; IVAC: infectious ventilator-associated condition; PVAP: possible ventilator-associated pneumonia; CDC: Centers for Disease Control; ANVISA: Agência Nacional de Vigilância Sanitária

**8 – SUPPLEMENTARY FIGURES**


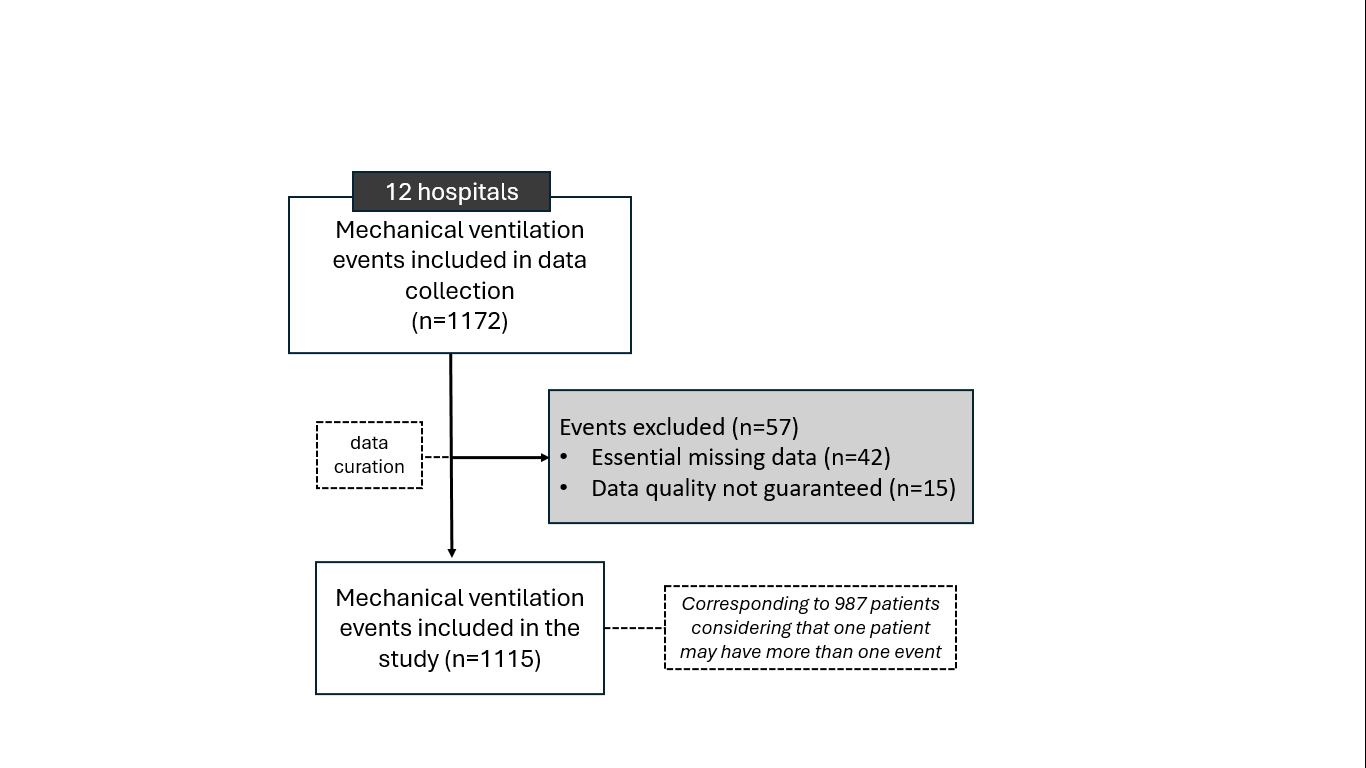


**Supplementary Figure 1** Flowchart of enrollment and exclusion details.


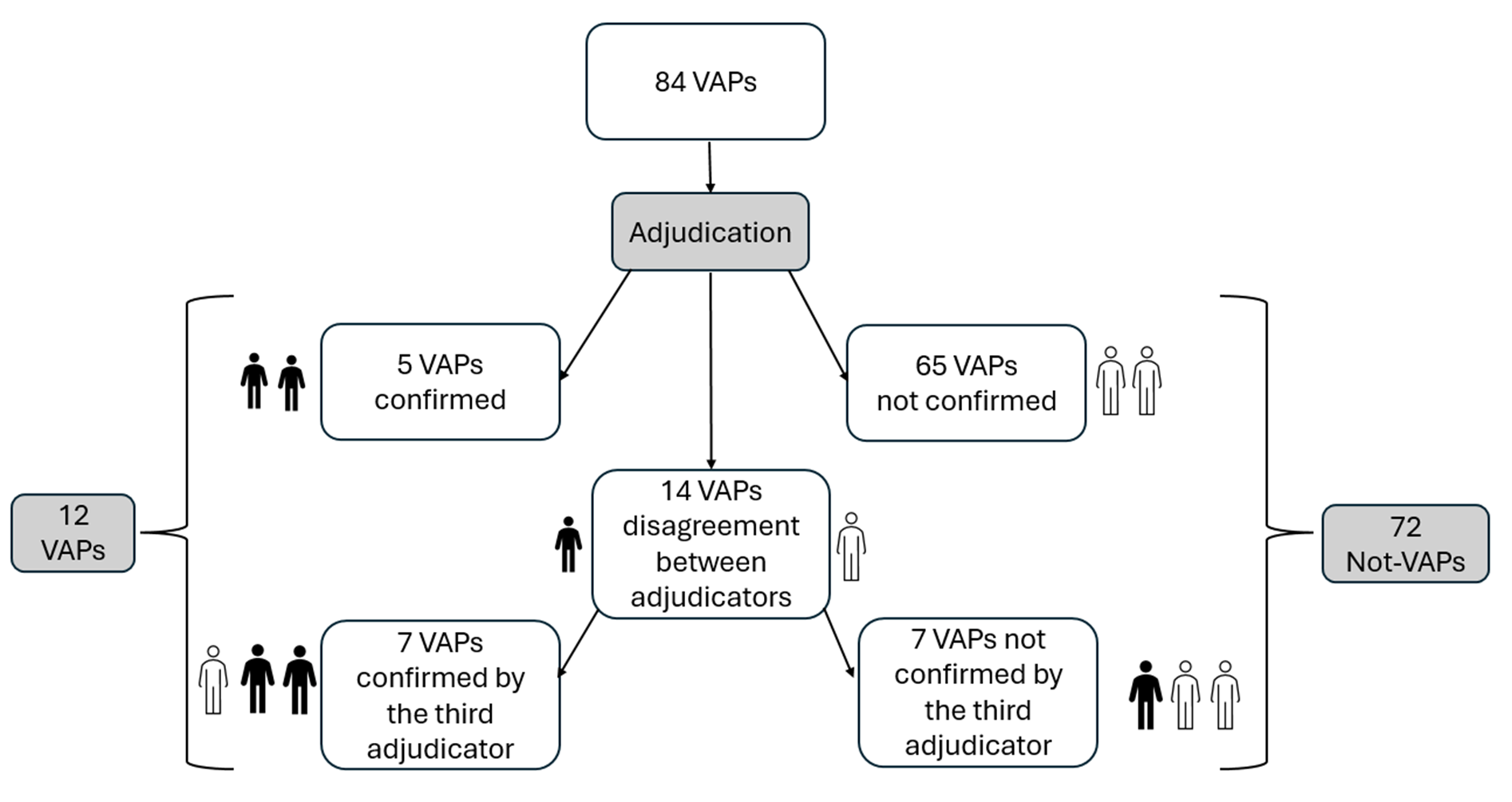


**Supplemetary Figure 2** VAP Adjudication (ANVISA criteria); VAP: ventilator associated pneumonia; ANVISA: Agência Nacional de Vigilância Sanitária.


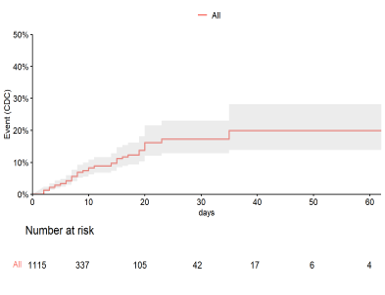


**Supplementary Figure 3** Kaplan-Meier analysis of VAP (CDC) incidence.

**9 - references**

1. Tomazini BM, Nassar AP Jr, Lisboa TC, Azevedo LCP de, Veiga VC, Catarino DGM, et al. IMPACTO-MR: um estudo brasileiro de plataforma nacional para avaliar infecções e multirresistência em unidades de terapia intensiva. Rev Bras Ter Intensiva. 2022;34:418-25.

2. Nascimento GM, Gomes Rodrigues DL, Mangas Catarino DG, Piastrelli FT, Cheno MY, Braz KCC, et al. Application of ventilator-associated events (VAE) in ventilator-associated pneumonia (VAP) notified in Brazil (IMPACTO MR-PAV): a protocol for a cohort study. BMJ Open. 2023;13:e076047.

3. NOTA TECNICA ANVISA - CRITERIOS DIAGNÓSTICOS DE IRAS[.](http://paperpile.com/b/wOQJDV/2R3Z) <https://www.gov.br/anvisa/pt-br/centraisdeconteudo/publicacoes/servicosdesaude/notas-tecnicas/notas-tecnicas-vigentes/nota-tecnica-no-03-2024-criterios-diagnosticos-de-iras/view.> [(accessed October 16, 2024).](http://paperpile.com/b/wOQJDV/2R3Z)

4. [for Disease Control C, Prevention, Others. CDC/NHSN surveillance definitions for specific types of infections. 2019.](http://paperpile.com/b/wOQJDV/8IPu)
